# Internal Medicine

**Published:** 1892-08

**Authors:** F. J. Thornbury


### Progress in Medical Science.

### INTERNAL MEDICINE.

Conducted by F. J. THORNBURY, M. D.

### CHLOROSIS.

Prof. Pick, of Prague, with his characteristic progressiveness in the line of original investigation, now believes the underlying causative influence in chlorosis to be an anto-intoxication from absorbed ptomaine products in the stomach. He, therefore, washes out the latter each morning, and then administers some preparation of iron. In three or four weeks marked improvement is noticeable, and this in cases which have resisted other treatment for months. Drachm doses of creasote in sugar of milk prevents the abnormal fermentation caused by the undigested particles of food where the irrigation referred to cannot be practised, although washing out the stomach is a simple procedure, and one easily executed.

Atonicity and dilatation, with anorexia, fetid breath, feeling of distention after ingestion of food, followed by emetations and vomiting, the picture assumed in many cases of chlorosis, are rational indications for this method of treatment.

There is found, further, hyperacidity, but diminution of hydrochloric acid ; the latter is supplied in the proportion of four parts of the pure acid to 1,000 of water, having the patient drink a glass of this solution after eating. This is Bouchard's method.

B-naplithal in doses of two grams, combined with two grams of salicylate of bismuth, are also efficient means of limiting the absorption of toxines from the gastric mucous membrane.

### CHLOROFORM IN TYPHOID FEVER.

Dr. Werner (*Medical Brief*,) has treated 130 cases of typhoid fever with drachm doses of a one per cent. solution of chloroform. The results are stated to be very satisfactory. This is in accordance with the now well recognized germicidal action of chloroform on the typhoid bacillus first noted experimentally by Behring. Drachm doses of the above solution every two hours, continued after subsidence of the fever, diminishes the severity of the symptoms, and reduces the liability to a relapse.

### DANGER OF HYPODERMIC SEPSIS.

For maintaining hypodermic needles pervious, and preventing them from rusting, the method recommended by Dr. T. M. Lawrence, (*N. Y. Medical Journal,*) that of filling the needle with vaseline and injecting any part of the latter which happens to remain after the piston is discharged, cannot meet with general approval, inasmuch as the vaseline, "harmlessly" injected, may contain bacteria.

Koch has long since demonstrated the impracticability of completely asepticising ointments and oils; many varieties of organisms may be cultivated from these media, permitting so imperfectly of dissemination of the antiseptic. The most efficient method of rendering the hypodermic needles and syringe sterile is to submerge them from three to five minutes in boiling water immediately before using. Then, to prevent rusting and maintain the needle pervious, dry heat should be applied, and the steel wire for the lumen should be passed through the flame.

---

### ULCERATION OF THE LARYNX FOLLOWING INTUBATION.

This liability constitutes the chief objection to this operation, but may be obviated to a large extent by daily *ex*tubation during continuance of the treatment, removing the tube for thirty minutes, or longer, in each twenty-four hours, the object being to asepticise the canula, and relieve the compression on the delicate mucous membrane.

The ulceration is largely a compression necrosis favored by aggregation of the pus formers at the point of insult.

The writer has seen demonstrated, post-mortem, seven cases of diphtheria and croup with ulceration on the anterior wall of the larynx. Two cases of recovery were observed in which, resulting from the subsequent cicatricial contraction, stenosis occurred, necessitating tracheotomy for relief of the dyspnea.

Wiederhoffer lays special stress on the importance of the recognition of this liability to laryngeal ulceration by physicians who intubate to the exclusion of other methods, tracheotomy, laryngotomy, and laryngo-tracheotomy respectively.

